# Analysis of virion associated host proteins in vesicular stomatitis virus using a proteomics approach

**DOI:** 10.1186/1743-422X-6-166

**Published:** 2009-10-12

**Authors:** Megan Moerdyk-Schauwecker, Sun-Il Hwang, Valery Z Grdzelishvili

**Affiliations:** 1 Department of Biology, University of North Carolina at Charlotte, Charlotte, NC 28223, USA; 2 Cannon Research Center, Carolinas Medical Center, Charlotte, NC 28203, USA

## Abstract

**Background:**

Vesicular stomatitis virus (VSV) is the prototypic rhabdovirus and the best studied member of the order *Mononegavirales*. There is now compelling evidence that enveloped virions released from infected cells carry numerous host (cellular) proteins some of which may play an important role in viral replication. Although several cellular proteins have been previously shown to be incorporated into VSV virions, no systematic study has been done to reveal the host protein composition for virions of VSV or any other member of *Mononegavirales*.

**Results:**

Here we used a proteomics approach to identify cellular proteins within purified VSV virions, thereby creating a "snapshot" of one stage of virus/host interaction that can guide future experiments aimed at understanding molecular mechanisms of virus-cell interactions. Highly purified preparations of VSV virions from three different cell lines of human, mouse and hamster origin were analyzed for the presence of cellular proteins using mass spectrometry. We have successfully confirmed the presence of several previously-identified cellular proteins within VSV virions and identified a number of additional proteins likely to also be present within the virions. In total, sixty-four cellular proteins were identified, of which nine were found in multiple preparations. A combination of immunoblotting and proteinase K protection assay was used to verify the presence of several of these proteins (integrin β1, heat shock protein 90 kDa, heat shock cognate 71 kDa protein, annexin 2, elongation factor 1a) within the virions.

**Conclusion:**

This is, to our knowledge, the first systematic study of the host protein composition for virions of VSV or any other member of the order *Mononegavirales*. Future experiments are needed to determine which of the identified proteins have an interaction with VSV and whether these interactions are beneficial, neutral or antiviral with respect to VSV replication. Identification of host proteins-virus interactions beneficial for virus would be particularly exciting as they can provide new ways to combat viral infections via control of host components.

## Background

The order *Mononegavirales *contains four families (*Rhabdoviridae, Paramyxoviridae, Filoviridae *and *Bornaviridae*), which include many lethal human pathogens (e.g. rabies, Ebola, and Hendra viruses); highly prevalent human pathogens, such as the respiratory syncytial and parainfluenza viruses; potential ethological agents of some neurobehavioral abnormalities and psychiatric disorders in humans (Borna disease virus); as well as viruses with a major economic impact on the poultry and cattle industries (e.g. Newcastle disease virus and rinderpest virus). All members of this order share a similar genome organization and common mechanisms of genome replication and gene expression, and, as with other RNA viruses with limited coding capacity, they exploit cellular proteins and pathways to facilitate many aspects of their replication cycle [1-3]. Identification of host-virus interactions can provide new insights into viral biology and developing new ways to combat viral infections via control of host components.

Vesicular stomatitis virus (VSV) is the best studied member of *Mononegavirales *and the prototypic rhabdovirus. There is now compelling evidence that enveloped virions (including members of *Mononegavirales*) released from infected cells carry numerous host (cellular) proteins some of which may play an important role in viral replication [4]. Several cellular proteins have been previously shown to be incorporated into VSV virions including tubulin [5], cyclophilin A [6], translation elongation factor 1 alpha (EF1a) [7], RNA guanylyltransferase [8], casein kinase II [9] and heat shock cognate 71 kDa protein (Hsc70, also known as HSPA8) [10]. However, to the best of our knowledge, no systematic study has been done to reveal the host protein composition for virions of VSV or any other member of *Mononegavirales*.

A proteomics approach utilizing mass spectrometry (MS) has been used to successfully identify cellular proteins in a number of enveloped viruses including poxviruses [11-13], herpesviruses [14-20], orthomyxoviruses [21], coronaviruses [22], and retroviruses [23-25]. Here we attempted the same strategy to identify cellular proteins within purified VSV virions, thereby creating a "snapshot" of one stage of virus/host interaction that can guide future experiments aimed at understanding molecular mechanisms of virus-cell interactions. Using this approach, we confirmed the presence of several previously-identified cellular proteins within VSV virions and identified a number of additional proteins.

## Results

### Purification of VSV from different cell types

**Figure 1:**
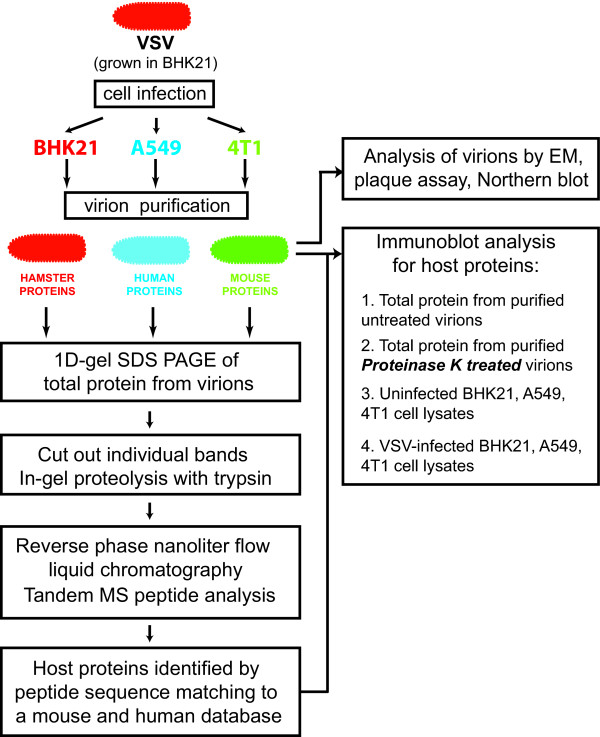
**Overview of the experimental procedure used in this study**.

**Figure 2:**
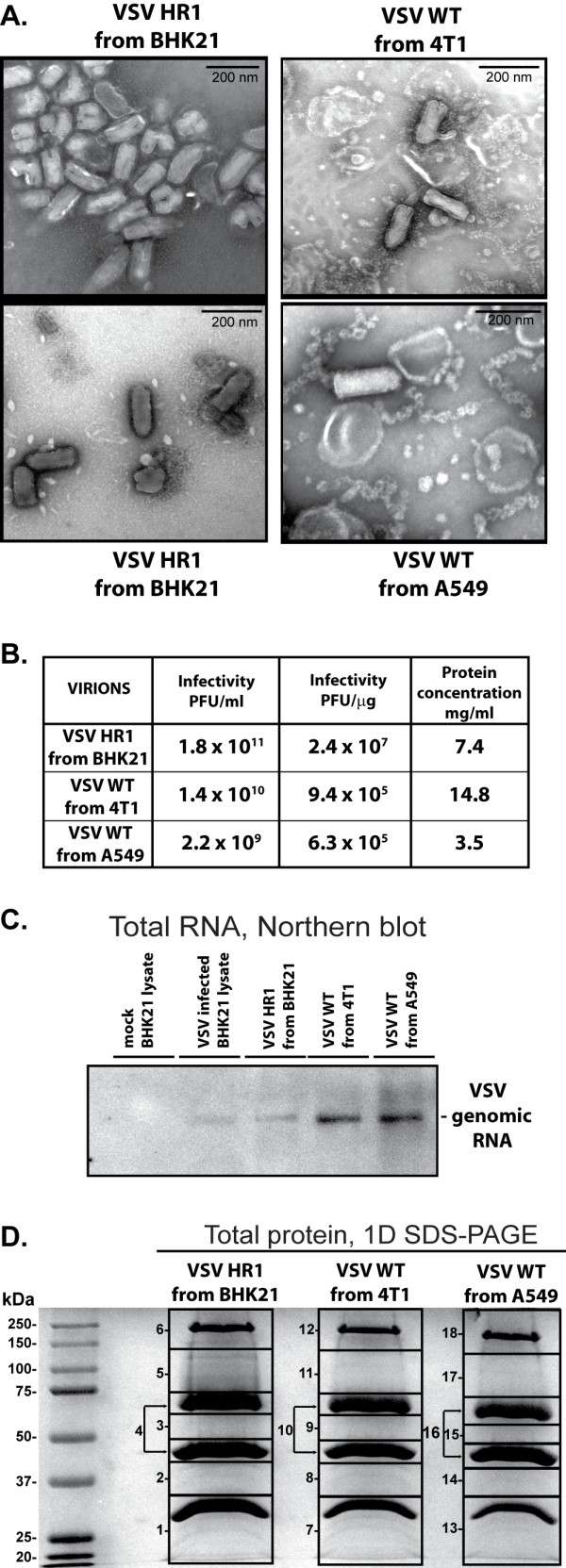
**Characterization of purified VSV virion preparations**. (A) Electron micrographs of the purified virion preparations from BHK21 (two different fields are shown), 4T1 and A549. Virions were absorbed to carbon-formvar coated grids and negatively stained with 2% uranyl acetate. (B) Infectivity (PFU/ml) of purified virions shown in (A) was calculated by standard plaque assay on BHK21 cells. Total protein concentration of these preparations was determined using a Bradford assay, and infectivity per total protein (PFU/μg) was calculated based on these two values. (C) Total RNA was extracted from uninfected (mock) or infected BHK21 cells or from purified virion samples containing 25 μg of protein, and analyzed by Northern blotting. RNA was separated on a 1.5% agarose-formaldehyde gel, transferred to a nylon membrane and detected using a probe complimentary to the 3' end of VSV genome. (D) 50 μg of total protein from each purified virion preparation was separated on a 10% SDS-PAGE gel, and stained with Coomassie Brilliant Blue R250. Numbered boxes indicate the gel segments cut out and analyzed by mass spectrometry.

Several cell lines [including BHK21 (hamster), HeLa (human), A549 (human), HEp2 (human), MIA PaCa (human), 4T1 (mouse), 3T3 (mouse), 3T10 (mouse), 2H-11 (mouse), MOVAS (mouse) and Vero (green monkey)] were tested for their ability to support robust replication of VSV and produce high titers of virus, which is required for successful purification and subsequent proteomic analysis. Based on this analysis (data not shown), we selected three cell lines, capable of producing the high VSV titers: BHK21 (baby hamster kidney cells), 4T1 (mouse mammary tumor cells) and A549 (human lung carcinoma cells) (Fig. 1). BHK21 has been extensively used as a standard cell line for growing VSV. A549 and 4T1 cells also supported suitable viral replication although to lower titers than BHK21 cells (Fig. 2B). The use of different cell lines allowed us to compare viral host protein content across species and cell types. In addition, the A549 and 4T1 cell lines were included to allow identification of cellular proteins potentially lacking sufficient homology to human or mouse proteins to be recognized from a hamster source (BHK21).

To grow and purify viruses, BHK21 cells were infected with VSV HR1 (Indiana serotype), while A549 and 4T1 cells were infected with VSV wild type (VSV-wt, Indiana serotype). VSV HR1 is a well characterized mRNA cap methylation defective VSV (Indiana serotype) host-range (hr) mutant which has a delay in replication but achieves wild type titers in BHK21 [26-29]. VSV HR1 was chosen for BHK21 infection as the milder cytopathic effect in BHK21 cells compared to VSV-wt aid exclusion of cellular debris (data not shown). However, VSV-wt was used for infection of A549 and 4T1 cells as replication of VSV HR1 was more inhibited in these two cell lines than in BHK21 due to its host-range growth phenotype (data not shown) and the cytopathic effect caused by VSV-wt in these cells was not as rapid or severe as seen with BHK21.

Virus containing media was collected at 20-28 hours (h) post infection (p.i.) when most cells were infected but significant cell detachment had not yet occurred (to maximize exclusion of cellular debris), and virions were purified using a discontinuous sucrose gradient purification protocol as described in the Materials and methods section. Initial concentration of virus by polyethylene glycol precipitation [30] as well as the use of continuous sucrose [30], cesium chloride [31], and iodixanol gradients [32] were also tried without significant improvement in sample infectivity or purity (data not shown).

Virion samples were examined by transmission electron microscopy (EM) for the presence of nonviral structures and to assess virion integrity. As shown in Figure 2A, the sample derived from BHK21 cells was primarily composed of particles readily recognizable as VSV although the possibility of some cellular contaminants cannot be ruled out. In addition to standard "bullet shaped" particles, there were also large numbers of "bent" particles where the virion appears to have been broken in half. These bent particles have been shown to be a substantial component of at least some VSV preparations and are infectious [31]. The presence of other "irregular" particles is consistent with previous studies demonstrating that VSV virions can easily undergo morphological changes when processed for visualization by EM [33,34]. In contrast to the BHK21 preparation, the virion preparations from A549 and 4T-1 showed fewer intact virus particles, and a large number of unwound nucleocapids could be seen (Fig. 2A). Some of the membranous structures present may represent the viral membranes dissociated from the nucleocapsids, although the possibility of them being cellular vesicles cannot be ruled out [35]. Consistent with the differences observed by EM (Fig. 2A) was the variation in the number of infectious particles per μg of total protein (Fig. 2B). However, when equal quantities of total protein were separated on a 10% SDS-PAGE gel and visualized by Coomassie staining (Fig. 2D) or analyzed by immunoblotting against VSV proteins (data not shown), the quantity and distribution of the viral proteins was similar in all samples as were the intensity and number of minor bands representing cellular or degraded viral proteins. Additionally, when total RNA extracted from samples containing equal quantities of protein was analyzed by Northern blotting using a probe complimentary to the 3' end of VSV genome, greater numbers of viral genomes were recovered from purified virus samples from 4T1 and A549 than from BHK21 cells (Fig. 2C). Smaller products representing defecting interfering genomic RNAs were not detected in any sample, even upon overexposure of the membrane (data not shown). This supports our hypothesis that the filament structures seen in Figure 2A for A549 and 4T1 virion preparations are unwound VSV nucleocapids containing viral genomic RNA. That these filaments are not cellular nucleic acids is also supported by the lack of detection of any histone or ribosomal proteins in the A549 and 4T1 samples by MS (Table 1). Together, the Northern blot (Fig. 2C) and Coomassie staining (Fig. 2D) data suggest the observed visual differences between samples (Fig. 2A) are at least in part due to differences in particle stability/infectivity rather than simply sample purity.

**Table 1:** Cellular proteins identified in purified virion preparations following 1-D SDS-PAGE and LC-MS/MS

				**Gel pieces found in ^d ^(No. of spectra/No. unique peptides)**			
					
**Protein name**	**Taxonomy ^a^**	**Accession No. ^b^**	**Mass (kDa)**	**BHK**	**4T1**	**A549**	**Other viruses found in ^e^**
Tubulin alpha	H	IPI00180675	50.1	3(11/9)			Influenza [21], HCMV [19], VV [11,13], HIV-1 [23], ASFV [58].
		
	M	IPI00110753	50.1	3(11/10)	9(2/2)	15(2/2)	

Annexin A2	H	IPI00455315	38.6	2(8/4)	8(4/3)	14(3/2)	Influenza [21], HCMV [19,59], VV [11], KSHV [20], HIV-1[23], HSV-1 [18], AlHV-1 [15].
		
	M	IPI00468203	38.5	2(12/7)	8(5/4)	14(2/2)	

Elongation factor 1-alpha	H	IPI00014424	50.5	3 (9/3)	9(9/4)		HIV-1 [23,24,60,61], VV [11,13], MCMV [17], HCMV [19], SARS-CoV [22].
		
	M	IPI00307837	50.3	3(9/3)	9(13/6)	15(3/2)	

Ubiquitin	H	IPI00719280	25.8	2(6/3),3(4/3), 4(3/2),5(3/2)	8(8/3),9(7/3), 11(2/2)	14(3/2), 17(2/2)	Influenza [21], HIV-1 [23,24,62], SIV [62], MMLV [25,62], VV [11,45], AcNPV [44], ASFV [45].
		
	M	IPI00923013	26.6	2(5/2),3(4/3), 4(3/2),5(3/2)	8(7/3),9(6/2), 10(2/2), 11(2/2)	14(3/2), 15(3/2), 17(2/2)	

Integrin beta-1	H	IPI00217561	91.7^c^	6(2/2)	12(3/2)	18(6/2)	Influenza [21], HIV-1 [23], MMLV [25].
		
	M	IPI00132474	88.2^c^	6(3/3)	12(4/3)		

Tubulin, beta	H	IPI00011654	49.7	3(11/7)	9(3/3)		Influenza [21], HCMV [19], EBV [16], VV [11,13], MMLV [25], ASFV [58].
		
	M	IPI00109061	49.9	3(9/6)	9(3/3)		

Actin, cytoplasmic	H	IPI00021439	41.7	4(3/2)	8(3/3)		Influenza [21], HCMV [19], EBV [16], VV [11,12,63], KSHV [20] MMLV [25], HIV-1 [23,39], MCMV [17], HSV-1 [18], ASFV [58], AlHV-1 [15], SeV [48], MV [49], RV [50].
		
	M	IPI00110850	41.7	4(3/2)	8(3/3)		

Elongation factor 2	M	IPI00466069	95.2	5(2/2)	11(3/2)		HIV-1 [23], KSHV [20], HCMV [19], SARS-CoV [22].

Transferrin receptor protein 1	H	IPI00022462	84.9^c^			17(3/2)	HSV-1 [18], VV [64].
		
	M	IPI00124700	85.7^c^		11(2/2)		

Low-density lipoprotein receptor-related protein 1, 85 kDa and 515 kDa subunits	H	IPI00020557	504.5	5(7/5), 6 (33/27)			
		
	M	IPI00119063	504.7	5(7/5), 6(38/29)			

Hsp90	H	IPI00784295	84.8	5(8/7)			HIV-1[23], VV [11], EBV [16], KSHV [20], HCMV [19], SARS-CoV [22].
		
	M	IPI00330804	84.6	5(8/8)			

Neural cell adhesion molecule 1	H	IPI00435020	93.3^c^	2(2/2), 6(7/6)			
		
	M	IPI00122971	119.3^c^	6(6/5)			

Heat shock cognate 71 kDa protein	H	IPI00003865	70.9	5 (8/6)			MMLV [25], HIV-1 [23,65], VV [11], RV [10], NDV [10], Influenza [10].
		
	M	IPI00323357	70.9	5(3/3)			

Chondroitin sulfate proteoglycan 4	H	IPI00019157	250.5	6(3/3)			
		
	M	IPI00128915	252.4	6(5/5)			

Prostaglandin F2 receptor negative regulator	H	IPI00022048	98.5^c^	6(4/4)			MMLV [25], HIV-1 [23].
		
	M	IPI00125497	106.0^c^	6(4/4)			

Enolase	H	IPI00216171	47.1	3(2/2)			Influenza [21], HCMV [19], EBV [16], KSHV [20], HIV-1 [23], VV [12].
		
	M	IPI00462072	47.0	3(4/4)			

Annexin A5	H	IPI00329801	35.8	2(5/3)			Influenza [21], HCMV [19], HIV-1[23], HSV-1 [18], VV [63].
		
	M	IPI00317309	35.7	2(4/2)			

Synaptic vesicle membrane protein VAT-1 homolog	H	IPI00156689	41.9	3(4/3)			
		
	M	IPI00126072	43.1	3(2/2)			

Annexin A4	M	IPI00353727	35.8	2(3/2)			Influenza [21].

ATP synthase alpha chain, mitochondrial	H	IPI00440493	59.7	3(2/2)			
		
	M	IPI00130280	59.7	3(2/2)			

ATP synthase beta chain, mitochondrial	M	IPI00468481	56.3	3(2/2)			HIV-1 [23], VV [63].

Casein kinase I	H	IPI00167096	39.1	2(2/2)			
		
	M	IPI00330729	38.9	2(2/2)			

CD44 antigen	H	IPI00297160	39.4^c^	5(3/2)			HIV-1 [23].
		
	M	IPI00223769	40.2^c^	5(2/2)			

Fascin	M	IPI00353563	54.3	3(2/2)			VV [12].

Fibronectin	M	IPI00113539	272.5	12(2/2)			HIV-1 [23].

Guanine nucleotide-binding protein G(o), alpha subunit 2	M	IPI00115546	39.9	2(3/2)			

Histone H3.2	M	IPI00230730	15.2	1(2/2)			

Histone H4	H	IPI00453473	11.2	1(2/2)			MMLV [25], HIV-1 [23], AlHV-1 [15], SARS-CoV [22].
		
	M	IPI00329998	11.4	1(2/2)			

Methyl-CpG-binding domain protein 4	M	IPI00321709	62.6	3(2/2)			

Monocyte differentiation antigen CD14	M	IPI00308990	39.2	3(3/2)			HIV-1 [23].

Pyruvate kinase	M	IPI00407130	58.0	3(2/2)			Influenza [21], KHSV [20], HIV-1 [23], VV [12,13], AlHV-1 [15].

Integrin alpha-3	M	IPI00126090	116.7^c^		12(7/5)		HIV-1 [23].

Annexin A3	M	IPI00132722	36.2		8(4/4)		

Envelope protein	M	IPI00406960	73.6		7(6/4)		

Basigin	M	IPI00113869	29.7		9(3/3)		HIV-1 [23].

EH-domain-containing protein 1	H	IPI00017184	60.6		9(3/3)		
		
	M	IPI00126083	60.6		9(3/3)		

Gag protein	M	IPI00224370	60.3		7(4/3)		

Lymphocyte antigen 74	M	IPI00115558	35.0		8(3/3)		

Tumor susceptibility gene 101 protein	H	IPI00018434	43.9		9(3/3)		HIV-1 [23].
		
	M	IPI00117944	44.1		9(3/3)		

Acid sphingomyelinase-like phosphodiesterase 3b	M	IPI00117534	51.6		9(2/2)		

Dystrophin	M	IPI00474450	425.8		8(2/2)		

Guanine nucleotide-binding protein G(I)/G(S)/G(T) beta subunit	H	IPI00003348	37.2		8(3/2)		
		
	M	IPI00116938	37.2		8(3/2)		

H-2 class I histocompatibility antigen, D-D alpha chain	M	IPI00110805	41.1		9(2/2)		

H-2 class I histocompatibility antigen, L-D alpha chain	M	IPI00109996	40.7		9(2/2)		

L-lactate dehydrogenase A chain	M	IPI00319994	36.3		8(2/2)		HCMV [19], VV [12], SARS-Cov [22].

Monocarboxylate transporter 1	M	IPI00137194	53.3		8(2/2)		

Myosin-9	H	IPI00019502	226.4		12(2/2)		VV [11], KHSV [20].

T-complex protein 1 subunit beta	M	IPI00320217	57.3		9(2/2)		HIV-1 [23].

Transmembrane protease, serine 11E	M	IPI00222870	50.0		9(2/2)		

Ubiquitin protein ligase E3 component n-recognin 4	M	IPI00378681	572.3		8(2/2)		

4F2 cell-surface antigen heavy chain	H	IPI00027493	57.9^c^			17(9/7)	HIV-1 [23].

Albumin	H	IPI00022434	71.7			14(3/3)	HIV-1 [23].

Annexin A1	H	IPI00218918	38.6			14(3/3)	Influenza [21], HCMV [19], VV [11], HIV-1[23], MCMV [17], HSV-1 [18], AlHV-1 [15].

CD109 antigen	H	IPI00152540	161.7			18(3/3)	

Transmembrane protein 2	H	IPI00170706	154.4			18(3/3)	

Aminopeptidase N	H	IPI00221224	109.4			18(2/2)	HIV-1 [23], HCMV [19].

Integrin alpha-V	M	IPI00319509	109.5			18(2/2)	HIV-1 [23].
		
	H	IPI00027505	116.0			18(2/2)	

Lutheran blood group glycoprotein	H	IPI00002406	67.4^c^			17(2/2)	

Neutral amino acid transporter B(0)	H	IPI00019472	56.6^c^			17(2/2)	

Programmed cell death 6 interacting protein	H	IPI00246058	96.8			17(2/2)	MMLV [25], HIV-1 [23].

Ras-related protein Rab-11B	M	IPI00135869	24.3			13(2/2)	

Ras-related protein Rap-1A	M	IPI00138406	21.0			13(2/2)	HIV-1 [23].

Solute carrier family 2, facilitated glucose transporter member 1	H	IPI00220194	54.1^c^			17(2/2)	
		
	M	IPI00308691	53.9^c^			17(2/2)	

T-complex protein 10a	M	IPI00122340	47.1			18 (3/2)	

^a ^From search of human (H) or mouse (M) database

^b ^International protein index accession numbers

^c ^Glycosylated protein

^d ^Gel bands were numbered as depicted in Figure 2D

^e ^ASFV, African swine fever virus; AlHV-1, Alcelaphine herpesvirus-1; AcNPV, Autographa californica nuclear polyhedrosis virus; EBV, Epstein-Barr virus; HCMV, human cytomegalovirus; HIV-1, human immunodeficiency virus-1; KSHV, Kaposi's sarcoma-associated herpesvirus; MV, measles virus, MMLV, Moloney murine leukemia virus; MCMV, murine cytomegalovirus; NDV, Newcastle disease virus; RV, rabies virus; SeV, Sendai virus; SARS-CoV, severe acute respiratory syndrome coronavirus; SIV, simian immunodeficiency virus; VV, vaccinia virus

### Identification of virion associated proteins using proteomic approach

For MS analysis, 50 μg of total protein was separated on a 1-D SDS-PAGE gel, stained with Coomassie Brilliant Blue R250 and gel bands were cut out for analysis as indicated in Figure 2D. Gel pieces were subjected to in-gel trypsin digestion and the resulting peptides were extracted from the gel matrix, separated using reverse-phase nano-liquid chromatography, analyzed by tandem MS [36], and searched against human and mouse databases (concatenated with a VSV protein database), as described in the Materials and methods section. All five VSV proteins were identified by this analysis, as well as 64 cellular proteins (Table 1) plus keratins (not shown in Table 1). Of the 64 proteins, nine were identified in more than one sample. Five proteins [tubulin alpha, annexin A2, EF1a, ubiquitin and integrin β1] were identified in all three samples while tubulin beta, cytoplasmic actin and translation elongation factor 2 were identified only in the BHK21 and 4T1 derived virion preparations; and transferin receptor protein 1 was identified only in the 4T1 and A549 derived preparations.

### Confirmation of virion incorporation for several cellular proteins

**Figure 3:**
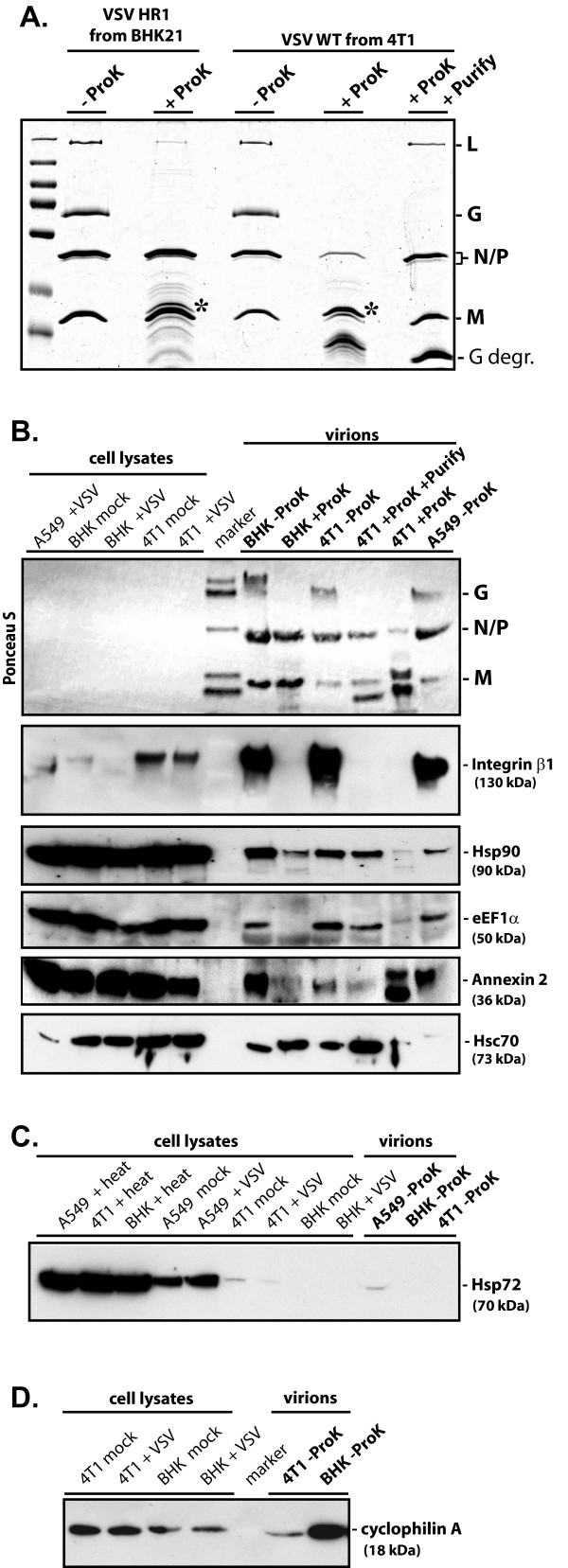
**Verification of protein incorporation within the virion preparations**. Purified virion preparations (shown in Figure 2A) were left untreated (-ProK) or were treated with Proteinase K (+ProK) for 1.5 h at 37°C to remove all surface exposed proteins. Following ProK treatment, one 4T1-derived virion sample was also centrifuged through a 20% sucrose gradient to remove ProK and free floating peptides (+ProK +Purify). (A) 5 μg of total protein from untreated purified virions or the viral protein equivalent from the ProK treated samples were separated on a 10% SDS-PAGE gel and stained with Coomassie Brilliant Blue R250. Asterisk indicates position of the ProK protein above the M protein. G degr., indicates VSV G protein fragment(s) generated as a result of ProK treatment. (B-D) Immunoblots were performed using 10 μg cellular lysate from mock infected or VSV-wt (+VSV) infected cells harvested at 18 h p.i. or 10 μg of heat shocked (+heat) cellular lysates harvested after a 4 h incubation at 43°C, and 50 μg of total protein from ProK treated or untreated purified virion preparations. Proteins were separated on gradient 8-16% (B and C) or 15% SDS-PAGE (D) gels, transferred to PVDF membranes and rapidly stained with the reversible dye Ponceau S prior to the use of antibodies to confirm levels of viral proteins (cellular proteins were not detectable). Primary antibodies were against integrin β1, heat shock protein 90 kDa (Hsp90), translation elongation factor 1 alpha (EF1α), annexin 2, heat shock cognate 70 kDa (Hsc70), stress-inducible 70 kDa heat shock protein (Hsp72), and cyclophilin A, as indicated.

Several proteins [integrin β1, heat shock protein 90 kDa (Hsp90), Hsc70, annexin 2, EF1a] identified by MS were picked for analysis by immunoblotting to confirm their presence in the virion preparations. 50 μg of purified virions and 10 μg of cellular lysate prepared from mock infected cells or cells infected with VSV-wt harvested at 18 h p.i. were separated on SDS-PAGE gels and analyzed by immunoblotting (Fig. 3B), as described in the Materials and methods section.

To determine whether these selected cellular proteins were incorporated within virions, a portion of purified virions from BHK21 and 4T1 cells were treated with proteinase K (ProK). ProK treatment degrades any proteins associated with the exterior surface of the virion as well as the exposed portion(s) of any membrane proteins, while the viral envelope excludes ProK from the interior of the virion, thereby protecting proteins incorporated into the virion [37,38]. A portion of the treated virions from 4T1 cells were additionally purified by centrifugation through a 20% sucrose cushion. This process removed the proteinase and cleaved peptides and aided removal of any residual contaminating vesicles as proteinase treatment would decrease the density of vesicles to a greater extent than the virions [39]. Due to insufficient quantities of purified virions from the preparations analyzed by MS, we were unable to conduct all treatments with preparations from all three cell lines.

As seen in Figures 3A and 3B, ProK treatment resulted in the almost complete removal of the viral glycoprotein (G). However, residual quantities of G could be visualized by immunoblotting with anti-VSV antibodies, indicating the process was not 100% efficient (data not shown). In contrast, substantial amounts of the viral matrix (M), nucleocapsid (N), phosphoprotein (P) and large polymerase (L) proteins were protected from ProK activity by the viral membrane, although we estimate that about 50% of these proteins were lost from the BHK21 derived virions during this process, about 70% from the 4T1 derived virions and about 90% from the ProK treated and additionally purified 4T1 derived virions. This loss of presumably membrane-protected proteins was likely due to physical disruption of the virion membrane, and the differences in protein loss were consistent with the varying degrees of virion disruption observed by EM (Fig. 2A). Due to the high loss of protein from the 4T1 derived sample, we were unable to obtain sufficiently concentrated ProK-treated virions derived from 4T-1 cells without additional purification, so slightly less than 50 μg of this virus was used for immunoblotting (Fig. 3A-B, sample 4T-1 + ProK).

The presence of integrin β1, Hsp90, Hsc70, annexin 2 and EF1a in the virion preparations was confirmed by immunoblotting. With the exception of integrin β1, all of the proteins could be detected in at least one of the ProK treated samples at levels similar to the untreated samples. The reason for the variable responses of the ProK treated samples is not known, however, the presence of a protein in the ProK treated and additionally purified VSV ("4T1+ProK+Purify" sample, Fig. 3A-B) would strongly indicate the presence of the protein within virions. This sample consistently showed a high level of protection of these proteins except for annexin 2 which can be located either intracellularly or on the extracellular membrane, and therefore a portion of it is likely vulnerable to ProK degradation [40]. In contrast, for integrin β1, where the extracellular domain is expected to be outside of the viral membrane, the protein levels were greatly reduced in all proteinase K treated samples, as seen for the viral G protein.

We also analyzed untreated virion samples for two proteins not detected by our MS analysis. The stress-inducible 70 kDa heat shock protein 70 (Hsp72, also known as Hsp70 and HSPA1A), previously shown to enhance measles virus transcription [41,42] and determine measles neurovirulence in mice [43], was readily detectable by immunoblotting in lysates prepared from cells heat shocked at 43°C for 4 h (Fig. 3C). Without heat shock, Hsp72 was also easily detected in A549 lysates, weakly detected in 4T-1 lysates and not detected at all in BHK21 lysates. In keeping with this, Hsp72 could be detected in virions from A549 cells but was not seen in virions from 4T1 or BHK21 cells. That Hsp72 was detected equally well in all the heat shocked lysates suggests these differences are not solely due to variable antibody recognition of Hsp72 from different sources. VSV infection did not appear to induce Hsp72 expression at the time point analyzed. Immunoblotting also confirmed the presence of cyclophilin A (Fig. 3D) in virions derived from BHK21 and 4T1 cells (the A549 preparation was not tested for this protein).

## Discussion

In this study, we conducted the first systematic study of the cellular protein composition of VSV virions. To compare host protein content of VSV virions across species and cell types, we analyzed virions isolated from three different cell types of human, mouse and hamster origin. In total, our analysis successfully identified all five VSV proteins as well as 64 cellular proteins (Table 1), plus keratins. For the majority of the identified proteins, the predicted molecular weight was consistent with the size range encompassed by the 1-D SDS-PAGE gel slice the protein was found in, which served as an additional confirmation of the cellular protein identity. However, the two groups of proteins, keratins (identified but not shown in Table 1) and ubiquitin (Table 1), were broadly distributed among the gel slices. Keratins are common environmental contaminants and their broad distribution among the gel slices without correlation to the predicted molecular weight suggests contamination as the primary source of the keratin peptides identified in our MS analysis. Ubiquitin was also detected across a wide range of molecular weights, suggesting that at least some viral and/or cellular proteins within VSV virions are ubiquitinated, although phospholipid anchored ubiquitin (not linked to any protein) has been found in the envelopes of several different viruses [44,45]. Further studies are needed to determine the functional role of ubiquitin association with VSV virions.

Of the 64 identified proteins, relatively few were identified in multiple samples originated from different cell sources. Five proteins [tubulin alpha, annexin A2, EF1a, ubiquitin and integrin β1] were identified in all three samples while tubulin beta, cytoplasmic actin and translation elongation factor 2 were identified only in the BHK21 and 4T1 derived virion preparations; and transferin receptor protein 1 was identified only in the 4T1 and A549 derived preparations. There are several possible explanations for this limited overlap including: (1) Limitations in the ability of MS to detect certain proteins, particularly those found in low abundance; (2) Differences in sample quality between the three preparations; (3) Cell specific differences in gene expression or virus assembly; (4) Some proteins of hamster origin (BHK21) potentially lack sufficient homology to be identified using the mouse or human databases used in this study.

Consistent with the first possibility, Hsp90 and Hsc70 were detected by MS only in the BHK21 derived virions. However, when examined by immunoblotting (Fig. 3B), Hsp90 was detected in all three virion preparations, and Hsc70 was detected in the BHK21 and 4T1 virion preparations (the antiserum did not react strongly with Hsc70 from A549, preventing any conclusion about this sample). Importantly, of the 64 proteins, 35 were identified on the basis of only two unique peptides while for other 14 proteins only three unique peptides were identified. All this suggests other proteins may also be present in multiple virion preparations despite being detected in only a single sample by MS.

In regard to the second possibility, the sample derived from BHK21 cells was primarily composed of particles readily recognizable as VSV, while the virion preparations from A549 and 4T-1 cells showed fewer intact virus particles and a large number of unwound VSV nucleocapids containing viral genomic RNA (Fig. 2A), and had a lower infectivity per μg of protein. Therefore, it is possible that some of the cellular proteins normally present within viral particles were "leaked out" during virus purification from A549 and 4T1 cells (accounting for their absence in those preparations). Furthermore, it is possible that some cellular proteins found in the A549 and 4T1 preparations are associated with free nucleocapsids rather than the virions. These differences in virion properties may have also impacted some of the other assays used in this study. For example, genome isolation may have been more efficient from the A549 and 4T1 samples, perhaps partially accounting for the higher number of genomes detected in these preparations. It may have also had an effect on the proteinase K assay as suggested by the fact that approximately 70% of the supposedly internal viral proteins were degraded when virions isolated from 4T1 cells were treated with proteinase K as opposed to approximately 50% for virions isolated from BHK21 cells.

The variability in proteins identified in the different virus preparations could also be due to cell specific differences in gene expression or virus assembly. An apparent example of the former is Hsp72 which has been shown to enhance measles virus transcription [41,42] and is a determinant of measles neurovirulence in mice [43]. This protein was not specifically detected by our MS analysis although the closely related and constitutively expressed Hsc70 protein was identified (Table 1 and Figure 3), as were several peptides common to both Hsc70 and Hsp72. Upon heat shock, Hsp72 could be detected by immunoblotting in lysates from all three cell types, but in the absence of this stress, was easily detectable in A549 lysates, weakly detected in 4T1 lysates and not detected at all in BHK21 lysates. In keeping with this expression profile, Hsp72 could be detected only in virions from A549, demonstrating that, at least in some cases, host cell protein expression may affect incorporation of cellular proteins into virions.

Using a MS approach, this study confirmed the presence of several cellular proteins within VSV virions (tubulin [5], translation elongation factor 1 alpha [7], and Hsc70 [10]). However, we were unable to detect at least three proteins previously shown to be associated with VSV virions (cyclophilin A [6], cellular RNA guanylyltransferase [8], and casein kinase II [9]) as well as several proteins shown to bind individual VSV proteins including the beta and gamma subunits of elongation factor 1 [7] and heat shock protein 60 (Hsp60) [46]. Failure of our analysis to detect some of these proteins does not challenge their potential role in VSV replication, as these proteins may be present within the virions but were not detectable in our MS analysis, or, in the case of the protein interactions shown outside the virion, the described interactions may be transient. When two of our virus preparations were tested for the presence of cyclophilin A by immunoblotting, it was detected (Fig. 3D), demonstrating the list of proteins generated by our MS analysis is not entirely inclusive.

Due to the nature of a MS proteomics approach, it will be necessary to confirm the presence of the identified proteins within the VSV virion and their role is viral replication, as incorporation within the virion does not necessarily imply a functional significance. "Accidental" incorporation of cellular proteins is particularly likely to occur during virus budding as abundant cytosolic proteins can be trapped by the newly forming viral envelope and host proteins are not excluded from the membrane used to form the envelope. We have initiated this process for several proteins of potential interest whose presence in the virion has not previously been shown (Hsp90, actin, annexin 2, and integrin β1), by confirming their presence in our virion preparations by immunoblotting, and, for the non-membrane proteins, confirming their presence within the virion by proteinase K protection assay (Fig. 3B and data not shown).

Pharmacological inhibition of Hsp90 or its knockdown by siRNA has been shown to inhibit replication of several negative-strand RNA viruses including VSV but its presence within VSV virions was not previously investigated [47]. Actin has previously been shown to be incorporated into Sendai [48], measles [49] and rabies [50] virions but was not detected in VSV virions [50]. Here, using MS, we have shown actin to be present within VSV as well (Table 1). Annexins bind phospholipids in a calcium dependent manner and are believed to help direct membrane-membrane and membrane cytoskeleton interactions. In particular, annexin 2 has been proposed to facilitate HIV-1 assembly at cellular membranes [23]. Integrin β1 forms heterodimers with various alpha integrins that function in both cell adhesion and cell signaling. While proteins found in multiple preparations may not be more important than those found only a single sample, the presence of annexin 2 and integrin β1 in all three samples as well as in a number of other viruses (for example, integrin β1 has also been detected in influenza, HIV-1 and Moloney murine leukemia virus), suggests that these two proteins may be involved in widely used viral processes (see far right column of Table 1 for complete listing and references). In fact, many of the cellular proteins identified in our study have been found in association with the virions of different RNA and DNA viruses (Table 1) suggesting that enveloped viruses may use similar cellular pathways for their assembly and exit from the cell. Currently, a similar study using a proteomics approach is being conducted in our laboratory to identify cellular proteins in virions of other members of the family *Mononegavirales*. Such comparative analysis will reveal how similar or different the cellular content of virions are among different members of this order.

## Conclusion

In summary, this is, to our knowledge, the first systematic study of the host protein composition for virions of VSV (or any other member of the order *Mononegavirales*). We have successfully used a proteomic approach to confirm the presence of several cellular proteins within VSV virions and to identify a number of additional proteins likely to also be present within the virions, some of which may play an important role in VSV replication and possibly be involved in previously unconsidered pathways in the virus life cycle. However, we recognize the potential of proteins not associated with virions to persist in our preparations despite purification and that these would also be identified by a global proteomics approach. Additionally, the inclusion of a protein within the virion does not necessarily imply a functional significance. Therefore, future experiments are needed to determine which of the identified proteins interact with VSV and whether these interactions are beneficial, neutral or antiviral with respect to VSV replication. Identification of host proteins-virus interactions beneficial for virus would be particularly exciting as they can provide new ways to combat viral infections via control of host components.

## Materials and methods

### Cells and viruses

The following cell lines were used in this study: Syrian golden hamster kidney fibroblast cells (BHK21; ATCC# CCL-10), mouse mammary gland adenocarcinoma cells (4T1; ATCC# CRL-2539), and human epithelial lung carcinoma cells (A549; ATCC# CCL-185). Monolayer cultures of these cell lines were maintained in Minimum Essential Medium (Eagle's MEM, Cellgro) or in Dulbecco's modified Eagle's medium (DMEM, Cellgro) supplemented with 9% fetal bovine serum (FBS, Gibco) in a 5% CO_2 _atmosphere at 37°C. Infectivity (PFU/ml) of virus stocks was calculated by standard plaque assay on BHK21 cells. To grow and purify viruses, cells were infected with wt or mutant VSV and incubated at 34°C. BHK21 cells were infected with VSV HR1 at a multiplicity of infection (MOI) of 0.005, while A549 and 4T1 cells were infected with VSV wild type (VSV-wt, Indiana serotype) at an MOI of 0.1 and 0.5, respectively. VSV HR1 is a well characterized mRNA cap methylation defective VSV (Indiana serotype) mutant [26,28,29] with a mutation in the L protein with a D to V substitution at position 1671 [26,27]. This mutation completely eliminates viral mRNA cap methylation at both the guanine-*N7 *and 2'-*O*-adenosine positions [26,27] and results in subsequent non-translatability of primary VSV transcripts in non-permissive cell lines [51-53]. As a result, VSV HR1 displays a host-range (hr) phenotype characterized by severely restricted growth in most cell types but only slightly delayed growth in a limited number of "permissive" cells including BHK21 cell line where it achieves wild type titers.

### Virus purification and protease treatment

Virus containing media was collected at 20-28 hours (h) post infection (p.i.) when most cells were infected but significant cell detachment had not yet occurred (to maximize exclusion of cellular debris). The media was centrifuged at 3000 × g for 10 minutes (min) to remove large cellular debris and then at 71,000 × g and 4°C for 1 h in a Beckman SW32 Ti rotor to pellet the viral particles. The viral pellet was resuspended in ET buffer (1 mM Tris-HCl pH 7.5, 1 mM EDTA) with 10% DMSO and centrifuged in a 7-60% discontinuous sucrose gradient composed of steps of 2 ml of 60% (w/w) sucrose, 3 ml of 45% sucrose, 4.5 ml of 25% sucrose and 1.5 ml of 7% sucrose. Sucrose solutions were made in HEN buffer (10 mM HEPES pH 7.4, 1 mM EDTA, 100 mM NaCl). Following centrifugation overnight at 130,000 × g and 4°C using a Beckman SW40 Ti rotor, the virus containing band was removed from the gradient and diluted with ET buffer. The virus was pelleted by centrifugation at 130,000 × g and 4°C for 1 h using a Beckman SW40 Ti rotor and resuspended in ET/DMSO buffer. Viral titers were determined by standard plaque assay on BHK21 cells.

For protease treatment, purified virions from BHK21 and 4T1 cells were treated with 0.08 μg proteinase K (ProK) per 1 μg total protein. After 1.5 h incubation at 37°C, phenylmethanesulphonylfluoride (PMSF) was added to a final concentration of 5 mM and the samples were incubated on ice for 15 min to stop proteinase activity. A portion of the treated virions from 4T1 cells were also centrifuged through a 2 ml 20% sucrose cushion at 173,000 × g and 4°C for 2.5 h using a Beckman SW40 Ti rotor. The pelleted virus was resuspended in ET buffer.

### Electron microscopy

Virions were absorbed to carbon-formvar coated grids (Electron Microscopy Sciences) by floating grids on 4 μl drops of sample for 1 min. Grids were blotted dry and stained with 2% uranyl acetate in water for 30 seconds. Excess stain was removed and the grids allowed to air dry. Samples were visualized using a Philips CM10 transmission electron microscope.

### Protein identification following 1D-SDS-PAGE

50 μg of total protein from each purified virion sample was separated on a 10% SDS-PAGE gel, stained with Coomassie Brilliant Blue R250 and gel bands were cut out for analysis. Gel pieces were subjected to in-gel trypsin digestion and the resulting peptides were extracted from the gel matrix, separated using reverse-phase nano-liquid chromatography and analyzed by tandem MS as described previously [36]. Briefly, samples were separated by a 68 min linear gradient from 90% Solvent I (0.1% formic acid in water)/Solvent II (0.1% formic acid in acetonitrile) to 50% Solvent I/II at a flow rate of 500 nl/min on reversed phase chromatography using a trap/elute method with a in-house C_18 _sample trap in line with a C_18 _analytical column. The spectra were searched using the SEQUEST algorithm of the Bioworks software (ThermoFisher, San Jose, CA; version SRF v. 3) against the IPI.HUMAN.v.3.18 and IPI.MOUSE.v.3.18 databases concatenated with a VSV protein database. A parent ion mass tolerance of 2.0 Da, fragment ion mass tolerance of 1.0 Da, and a 16 Da differential modification for methionine oxidation were used for search parameters. Protein identifications were accepted when the peptide probability was greater than 95.0% [54], the protein probability was greater than 99.0%, and contained at least 2 identified peptides. Scaffold software was used for data compiling of each group and calculating spectral count [55-57].

### Northern blot analysis

Total RNA was extracted from purified virion samples containing 25 μg of protein using the QIAamp viral RNA extraction kit (Qiagen), ethanol precipitated and resuspended in 30 μl of RNase-free water. Half of this material was separated on a 1.5% agarose-formaldehyde gel and transferred to a nylon membrane. Following blocking with hybridization buffer (Ambion) at 45°C, the membrane was incubated overnight at 45°C in hybridization buffer containing 30 ng/ml of a 5'-biotinylated oligonucleotide complimentary to the first 55 nucleotides of the VSV genome (5'-biotin-GATCCTTAAGACCCTCTTGTGGTTTTTATTTTTTATCTGGTTTTGTGGTCTTCGT-3'). Following blocking with TBS with 0.1% Tween 20 and 1% non-fat milk powder at room temperature for 30 min, the membrane was incubated with a streptavidin-horseradish peroxidase-conjugate in that same buffer for 1 h. The Enhanced Chemiluminescence Plus (ECL+) protein detection system (GE Healthcare) was used for detection and the membrane was exposed to BioMax Light film (Kodak).

### Protein gel electrophoresis and immunoblot analysis

Cellular lysates were prepared by mock infecting 4T-1, A549 and BHK cells or by infecting them with VSV-wt at MOIs of 0.5, 0.1 and 0.05 respectively. Cells were harvested at 18 h p.i. and lysed in RIPA buffer (25 mM Tris-HCl pH 7.6, 150 mM NaCl, 1% NP-40, 1% Sodium deoxycholate and 0.1% SDS). For heat shocked lysates, uninfected cells were incubated at 43°C for 4 h and immediately harvested. Protein concentrations of cellular lysates and purified virions were determined by Bradford assay. 50 μg of purified virions and 10 μg of cellular lysates were separated on gradient 8-16% or 15% SDS-PAGE gels, transferred to PVDF membranes and rapidly stained with the reversible dye Ponceau S prior to the use of antibodies to confirm levels of viral proteins and the quality of protein transfer from gel to membrane. Membranes were blocked in TBS (0.5 M NaCl, 20 mM Tris pH 7.5) with 0.1% Tween 20 and 5% non-fat milk powder and then probed with antibodies against integrin β1 (N-20; Santa Cruz Biotechnology), Hsp90 (68; BD Bioscience), EF1a (D-15; Santa Cruz Biotechnology), annexin 2 (H-50; Santa Cruz Biotechnology), Hsc70 (1B5; Assay Designs), Hsp72 (C92F3A-5; Assay Designs), or cyclophilin A (2175; Cell Signaling Technology). Detection was with species specific horseradish peroxidase-conjugated secondary antibodies using the Enhanced Chemiluminescence Plus (ECL+) protein detection system (GE Healthcare) and exposure to BioMax Light film (Kodak).

## Competing interests

The authors declare that they have no competing interests.

## Authors' contributions

MM conducted all experiments except for the MS analysis. SIH provided virion MS analysis and sequence matching to mouse and human databases. MM drafted the manuscript. MM and VZG edited the manuscript. VZG provided overall supervision, financial support and prepared the final version of the manuscript. All authors read and approved the final manuscript.

## References

[B1] Lamb RA, Parks GD, Knipe DM, Howley PM (2007). Paramyxoviridae: The viruses and their replication. Fields Virology.

[B2] Lyles DS, Rupprecht CE, Knipe DM, Howley PM (2007). Rhabdoviridae. Fields Virology.

[B3] Whelan SP, Barr JN, Wertz GW (2004). Transcription and replication of nonsegmented negative-strand RNA viruses. Curr Top Microbiol Immunol.

[B4] Cantin R, Methot S, Tremblay MJ (2005). Plunder and stowaways: incorporation of cellular proteins by enveloped viruses. J Virol.

[B5] Moyer SA, Baker SC, Lessard JL (1986). Tubulin: a factor necessary for the synthesis of both Sendai virus and vesicular stomatitis virus RNAs. Proc Natl Acad Sci USA.

[B6] Bose S, Mathur M, Bates P, Joshi N, Banerjee AK (2003). Requirement for cyclophilin A for the replication of vesicular stomatitis virus New Jersey serotype. J Gen Virol.

[B7] Das T, Mathur M, Gupta AK, Janssen GM, Banerjee AK (1998). RNA polymerase of vesicular stomatitis virus specifically associates with translation elongation factor-1 alphabetagamma for its activity. Proc Natl Acad Sci USA.

[B8] Gupta AK, Mathur M, Banerjee AK (2002). Unique capping activity of the recombinant RNA polymerase (L) of vesicular stomatitis virus: association of cellular capping enzyme with the L protein. Biochem Biophys Res Commun.

[B9] Gupta AK, Das T, Banerjee AK (1995). Casein kinase II is the P protein phosphorylating cellular kinase associated with the ribonucleoprotein complex of purified vesicular stomatitis virus. J Gen Virol.

[B10] Sagara J, Kawai A (1992). Identification of heat shock protein 70 in the rabies virion. Virology.

[B11] Chung CS, Chen CH, Ho MY, Huang CY, Liao CL, Chang W (2006). Vaccinia virus proteome: identification of proteins in vaccinia virus intracellular mature virion particles. J Virol.

[B12] Manes NP, Estep RD, Mottaz HM, Moore RJ, Clauss TR, Monroe ME, Du X, Adkins JN, Wong SW, Smith RD (2008). Comparative proteomics of human monkeypox and vaccinia intracellular mature and extracellular enveloped virions. J Proteome Res.

[B13] Resch W, Hixson KK, Moore RJ, Lipton MS, Moss B (2007). Protein composition of the vaccinia virus mature virion. Virology.

[B14] Bortz E, Whitelegge JP, Jia Q, Zhou ZH, Stewart JP, Wu TT, Sun R (2003). Identification of proteins associated with murine gammaherpesvirus 68 virions. J Virol.

[B15] Dry I, Haig DM, Inglis NF, Imrie L, Stewart JP, Russell GC (2008). Proteomic analysis of pathogenic and attenuated alcelaphine herpesvirus 1. J Virol.

[B16] Johannsen E, Luftig M, Chase MR, Weicksel S, Cahir-McFarland E, Illanes D, Sarracino D, Kieff E (2004). Proteins of purified Epstein-Barr virus. Proc Natl Acad Sci USA.

[B17] Kattenhorn LM, Mills R, Wagner M, Lomsadze A, Makeev V, Borodovsky M, Ploegh HL, Kessler BM (2004). Identification of proteins associated with murine cytomegalovirus virions. J Virol.

[B18] Loret S, Guay G, Lippe R (2008). Comprehensive characterization of extracellular herpes simplex virus type 1 virions. J Virol.

[B19] Varnum SM, Streblow DN, Monroe ME, Smith P, Auberry KJ, Pasa-Tolic L, Wang D, Camp DG, Rodland K, Wiley S (2004). Identification of proteins in human cytomegalovirus (HCMV) particles: the HCMV proteome. J Virol.

[B20] Zhu FX, Chong JM, Wu L, Yuan Y (2005). Virion proteins of Kaposi's sarcoma-associated herpesvirus. J Virol.

[B21] Shaw ML, Stone KL, Colangelo CM, Gulcicek EE, Palese P (2008). Cellular proteins in influenza virus particles. PLoS Pathog.

[B22] Neuman BW, Joseph JS, Saikatendu KS, Serrano P, Chatterjee A, Johnson MA, Liao L, Klaus JP, Yates JR, Wuthrich K (2008). Proteomics analysis unravels the functional repertoire of coronavirus nonstructural protein 3. J Virol.

[B23] Chertova E, Chertov O, Coren LV, Roser JD, Trubey CM, Bess JW, Sowder RC, Barsov E, Hood BL, Fisher RJ (2006). Proteomic and biochemical analysis of purified human immunodeficiency virus type 1 produced from infected monocyte-derived macrophages. J Virol.

[B24] Saphire AC, Gallay PA, Bark SJ (2006). Proteomic analysis of human immunodeficiency virus using liquid chromatography/tandem mass spectrometry effectively distinguishes specific incorporated host proteins. J Proteome Res.

[B25] Segura MM, Garnier A, Di Falco MR, Whissell G, Meneses-Acosta A, Arcand N, Kamen A (2008). Identification of host proteins associated with retroviral vector particles by proteomic analysis of highly purified vector preparations. J Virol.

[B26] Grdzelishvili VZ, Smallwood S, Tower D, Hall RL, Hunt DM, Moyer SA (2005). A single amino acid change in the L-polymerase protein of vesicular stomatitis virus completely abolishes viral mRNA cap methylation. J Virol.

[B27] Grdzelishvili VZ, Smallwood S, Tower D, Hall RL, Hunt DM, Moyer SA (2006). Identification of a new region in the vesicular stomatitis virus L polymerase protein which is essential for mRNA cap methylation. Virology.

[B28] Horikami SM, De Ferra F, Moyer SA (1984). Characterization of the infections of permissive and nonpermissive cells by host range mutants of vesicular stomatitis virus defective in RNA methylation. Virology.

[B29] Simpson RW, Obijeski JF (1974). Conditional lethal mutants of vesicular stomatitis virus. I. Phenotypic characterization of single and double mutants exhibiting host restriction and temperature sensitivity. Virology.

[B30] McSharry JJ, Wagner RR (1971). Lipid composition of purified vesicular stomatitis viruses. J Virol.

[B31] McCombs RM, Melnick MB, Brunschwig JP (1966). Biophysical studies of vesicular stomatitis virus. J Bacteriol.

[B32] Finke S, Brzozka K, Conzelmann KK (2004). Tracking fluorescence-labeled rabies virus: enhanced green fluorescent protein-tagged phosphoprotein P supports virus gene expression and formation of infectious particles. J Virol.

[B33] Brouillette CG, Compans RW, Brandts JF, Segrest JP (1982). Structural domains of vesicular stomatitis virus. A study by differential scanning calorimetry, thermal gel analysis, and thermal electron microscopy. J Biol Chem.

[B34] Orenstein J, Johnson L, Shelton E, Lazzarini RA (1976). The shape of vesicular stomatitis virus. Virology.

[B35] Arstila P (1974). Characteristics of vesicular stomatitis virus envelopes released with saponin. J Gen Virol.

[B36] Hwang SI, Lundgren DH, Mayya V, Rezaul K, Cowan AE, Eng JK, Han DK (2006). Systematic characterization of nuclear proteome during apoptosis: a quantitative proteomic study by differential extraction and stable isotope labeling. Mol Cell Proteomics.

[B37] Denard J, Rundwasser S, Laroudie N, Gonnet F, Naldini L, Radrizzani M, Galy A, Merten OW, Danos O, Svinartchouk F (2009). Quantitative proteomic analysis of lentiviral vectors using 2-DE. Proteomics.

[B38] Betakova T, Nermut MV, Hay AJ (1996). The NB protein is an integral component of the membrane of influenza B virus. J Gen Virol.

[B39] Ott DE, Coren LV, Kane BP, Busch LK, Johnson DG, Sowder RC, Chertova EN, Arthur LO, Henderson LE (1996). Cytoskeletal proteins inside human immunodeficiency virus type 1 virions. J Virol.

[B40] Gerke V, Moss SE (2002). Annexins: from structure to function. Physiol Rev.

[B41] Parks CL, Lerch RA, Walpita P, Sidhu MS, Udem SA (1999). Enhanced measles virus cDNA rescue and gene expression after heat shock. J Virol.

[B42] Vasconcelos DY, Cai XH, Oglesbee MJ (1998). Constitutive overexpression of the major inducible 70 kDa heat shock protein mediates large plaque formation by measles virus. J Gen Virol.

[B43] Carsillo T, Traylor Z, Choi C, Niewiesk S, Oglesbee M (2006). hsp72, a host determinant of measles virus neurovirulence. J Virol.

[B44] Guarino LA, Smith G, Dong W (1995). Ubiquitin is attached to membranes of baculovirus particles by a novel type of phospholipid anchor. Cell.

[B45] Webb JH, Mayer RJ, Dixon LK (1999). A lipid modified ubiquitin is packaged into particles of several enveloped viruses. FEBS Lett.

[B46] Qanungo KR, Shaji D, Mathur M, Banerjee AK (2004). Two RNA polymerase complexes from vesicular stomatitis virus-infected cells that carry out transcription and replication of genome RNA. Proc Natl Acad Sci USA.

[B47] Connor JH, McKenzie MO, Parks GD, Lyles DS (2007). Antiviral activity and RNA polymerase degradation following Hsp90 inhibition in a range of negative strand viruses. Virology.

[B48] Lamb RA, Choppin PW (1978). Determination by peptide mapping of the unique polypeptides in Sendai virions and infected cells. Virology.

[B49] Tyrrell DL, Norrby E (1978). Structural polypeptides of measles virus. J Gen Virol.

[B50] Naito S, Matsumoto S (1978). Identification of cellular actin within the rabies virus. Virology.

[B51] Hercyk N, Horikami SM, Moyer SA (1988). The vesicular stomatitis virus L protein possesses the mRNA methyltransferase activities. Virology.

[B52] Horikami SM, Curran J, Kolakofsky D, Moyer SA (1992). Complexes of Sendai virus NP-P and P-L proteins are required for defective interfering particle genome replication in vitro. J Virol.

[B53] Horikami SM, Moyer SA (1982). Host range mutants of vesicular stomatitis virus defective in in vitro RNA methylation. Proc Natl Acad Sci USA.

[B54] Keller A, Nesvizhskii AI, Kolker E, Aebersold R (2002). Empirical statistical model to estimate the accuracy of peptide identifications made by MS/MS and database search. Anal Chem.

[B55] Sadygov RG, Eng J, Durr E, Saraf A, McDonald H, MacCoss MJ, Yates JR (2002). Code developments to improve the efficiency of automated MS/MS spectra interpretation. J Proteome Res.

[B56] Yates JR, Eng JK, McCormack AL (1995). Mining genomes: correlating tandem mass spectra of modified and unmodified peptides to sequences in nucleotide databases. Anal Chem.

[B57] Yates JR, McCormack AL, Link AJ, Schieltz D, Eng J, Hays L (1996). Future prospects for the analysis of complex biological systems using micro-column liquid chromatography-electrospray tandem mass spectrometry. Analyst.

[B58] Esteves A, Marques MI, Costa JV (1986). Two-dimensional analysis of African swine fever virus proteins and proteins induced in infected cells. Virology.

[B59] Wright JF, Kurosky A, Pryzdial EL, Wasi S (1995). Host cellular annexin II is associated with cytomegalovirus particles isolated from cultured human fibroblasts. J Virol.

[B60] Ott DE, Coren LV, Johnson DG, Kane BP, Sowder RC, Kim YD, Fisher RJ, Zhou XZ, Lu KP, Henderson LE (2000). Actin-binding cellular proteins inside human immunodeficiency virus type 1. Virology.

[B61] Cimarelli A, Luban J (1999). Translation elongation factor 1-alpha interacts specifically with the human immunodeficiency virus type 1 Gag polyprotein. J Virol.

[B62] Ott DE, Coren LV, Copeland TD, Kane BP, Johnson DG, Sowder RC, Yoshinaka Y, Oroszlan S, Arthur LO, Henderson LE (1998). Ubiquitin is covalently attached to the p6Gag proteins of human immunodeficiency virus type 1 and simian immunodeficiency virus and to the p12Gag protein of Moloney murine leukemia virus. J Virol.

[B63] Jensen ON, Houthaeve T, Shevchenko A, Cudmore S, Ashford T, Mann M, Griffiths G, Krijnse Locker J (1996). Identification of the major membrane and core proteins of vaccinia virus by two-dimensional electrophoresis. J Virol.

[B64] Vanderplasschen A, Mathew E, Hollinshead M, Sim RB, Smith GL (1998). Extracellular enveloped vaccinia virus is resistant to complement because of incorporation of host complement control proteins into its envelope. Proc Natl Acad Sci USA.

[B65] Gurer C, Cimarelli A, Luban J (2002). Specific incorporation of heat shock protein 70 family members into primate lentiviral virions. J Virol.

